# Optic Nerve Sonography in the Diagnostic Evaluation of Pseudopapilledema and Raised Intracranial Pressure: A Cross-Sectional Study

**DOI:** 10.1155/2015/146059

**Published:** 2015-03-22

**Authors:** Masoud Mehrpour, Fatemeh Oliaee Torshizi, Shooka Esmaeeli, Salameh Taghipour, Sahar Abdollahi

**Affiliations:** ^1^Department of Neurology and Stroke Center, Firoozgar General Hospital, Iran University of Medical Sciences, Tehran 1449614535, Iran; ^2^Iran University of Medical Sciences, Tehran 1449614535, Iran; ^3^Students' Scientific Research Center (SSRC), Tehran University of Medical Sciences, Tehran 1417755331, Iran

## Abstract

*Introduction*. Differentiating pseudopapilledema from papilledema which is optic disk edema and a result of increased ICP (intracranial pressure) is important and can be done with noninvasive methods like orbital ultrasound examination.* Method*. This was a cross-sectional study in which patients with optic nerve head swelling were referred for LP exam after optic nerve head swelling diagnosis confirmation and having normal brain imaging (CT scan). Before LP (lumbar puncture) exam the patients were referred for optic nerve ultrasound test of both eyes.* Results*. Considering 5.7 mm as the upper limit for normal ONSD (optic nerve sheath diameter), sensitivity and negative predictive value of optic sonography in diagnosis of pseudopapilledema are 100% for both eyes. Calculated accuracy validity of ONSD measurement in detecting pseudopapilledema is 90% for the right eye and 87% for the left eye.* Conclusion*. Our study demonstrated a close correlation between optic nerve sheath dilation on ocular ultrasound and evidence of elevated ICP with optic disk swelling. With the aid of noninvasive diagnostic tests we can avoid unnecessary concerns along with expensive and invasive neurological investigations while targeting the correct diagnosis in bilateral optic disk swelling. Our study showed optic nerve sonography as a reliable diagnostic method for further usage.

## 1. Introduction

While papill edema is Optic Nerve Head (ONH) edema secondary to increased intracranial pressure (ICP), pseudopapilledema is apparent ONH swelling that stimulates some features of papill edema but is secondary to an underlying, usually benign, process which can be congenital anomalies associated with the disk elevation, hyperopic disk, or ONH drusen.

Acquired disk edema includes papilledema as well as other causes of optic disk edema such as optic neuritis, anterior ischemic optic neuropathy, malignant hypertension, infiltrative optic neuropathies, and compressive optic neuropathy [1].

ONH drusen accounts for 75% of clinical cases of pseudopapilledema, occurs in up to 2% of general population, and is congenitally inherited; it has the same prevalence between men and women and is usually bilateral. Patients with ONH drusen are usually asymptomatic but visual field defects can be present.

Diagnosis is most reliably made by orbital ultrasound examination [1].

Clinical features in pseudopapilledema differ from papilledema; pseudopapilledema patients usually have no visual symptoms while papilledema patients can have the following symptoms: transient visual obscurations, blurring of vision, diplopia, decreased color perception, and so forth. Also papilledema is almost always bilateral while pseudopapilledema can be either bilateral or unilateral. Furthermore there are some funduscopic assessment features seen in true papilledema which can help to distinguish it from pseudopapilledema, such as hyperemia of the optic disk with surface telangiectatic vessels, congested vasculature, and associated flame hemorrhages, optic disk elevation, and blurring of the disk margin associated with obscuration of the retinal vessels that traverse it. Lack of spontaneous venous pulsations at disk margin suggests true papilledema, but it is not diagnostic [1].

ONH elevation tends to be the most intimidating ocular finding especially when it presents bilaterally. The foremost clinical aim is to differentiate pseudopapilledema from acquired disk edema. Papilledema is ONH edema as a result of increased ICP, which bears specific etiologic implications. The most important entity to consider in cases of increased ICP is a space occupying lesion of the brain. A through history and a dilated fundus examination with use of current diagnostic technologies can facilitate the diagnosis. Orbital ultrasound examination is reported to be a useful noninvasive way which increase our ability to diagnose and manage these challenging case scenarios [2–5].

The purpose of this paper is to provide clinical strategies that will enhance clinicians' assessment of bilateral disk elevations. In addition, this topic is important to review because effective management will reduce over referrals for neurological evaluations, thus decreasing health care costs while avoiding LP, which is the gold standard test for ICP measuring, and other expensive imaging technologies.

## 2. Method

This was a cross-sectional study in 2013-2014, in which 32 (64 eyes) patients with bilateral ONH swelling whom had visited the ophthalmology or neurology clinic of Firoozgar Hospital, located in Tehran, Iran, were included. We excluded patients who had contraindication for LP such as increased intracranial pressure due to brain mass, bleeding diathesis such as coagulopathy and thrombocytopenia, skin infection at puncture site, sepsis, abnormal respiratory pattern, focal neurologic deficit, and loss of consciousness. Ophthalmic evaluation was done by expert ophthalmologist to rule out other causes. Also computerized tomography (CT) scan was done for all of the patients; if it did show any mass or abnormality which could be the reason for optic disk edema, the patient must be excluded.

We explained the process of our research for all included patients before starting data gathering. We asked them to sign written testimonials if they accept being included; also they had the choice to stop cooperating with our project whenever they want. Furthermore we have to point that our study was noninvasive and did not harm any body and we respected Helsinki declaration all along our project. We obtained ethical approval and it was not a part of routine care.

The patients were referred for LP exam after ONH swelling diagnosis confirmation and normal brain imaging. Before LP exam we had measured the vertical and horizontal diameters of the optic nerves of both eyes by ultrasonography (US) in supine position. The probe was placed on the superior and lateral aspect of the orbit against the upper eyelid with the eye closed and angled slightly caudally and medially until the optic nerve was visualized as a linear hypoechoic structure with clearly defined margins posterior to the globe. The probe was always placed gently on the closed eyelid without any contact with the cornea or sclera. Contact with the eye was gentle and pressure never directly applied on the globe with the probe, as this can theoretically result in nausea, vomiting, and a vagal response. The ONSD was measured 3 mm behind the retina; the measurements were done by wetting the closed eyelids and using a 7.5 MHz linear probe. All of the measurement in optic sonography was done by expert and particular person. We compare the ultrasound results with the LP results as the gold standard of measuring ICP for each patient.

*Statistical Analysis*. All analysis and comparisons were done with SPSS version 16. The mean of right and left eyes ONSD (optic nerve sheath diameter), OND (optic nerve diameter), and CSF pressure were calculated. A receiver operating characteristic (ROC) curve was constructed to determine the optimal ONSD and OND cut-off to detect ICP > 20 mmHg. We calculated the sensitivity and specificity of this cut-off with 95% confidence intervals for the detection of ICP > 20 mmHg. Also the sensitivity, specificity, positive predictive value, negative predictive value, and accuracy validity of optic sonography were calculated for detecting of pseudopapilledema. At last we calculate the diagnostic accuracy which is in fact a criterion that considers sensitivity and specificity together for determination of optic sonography value in diagnosis of pseudopapilledema.

## 3. Results

We performed ocular sonography on 29 female and 3 male patients with swelled optic disk. The mean age of patients was 35.44 ± 13 (19–75 years old). 19 patients were below 35 years old and 13 were older than 35 (Table 1).

According to the literatures we set 5.7 mm and 20 mmHg as the cut-off values for ONSD and CSF pressure, respectively. We measured CSF pressure, RT.ONSD, and LT.ONSD in patients. Results show that 68.8% (22 patients) of the patients had CSF pressure more than 20 mmHg, and for 81.4% (26 patients) and (25 patients) 78% of patients RT.ONSD and LT.ONSD were more than the chosen threshold, respectively.

Due to our measurements, patients with high ICP had significantly larger ONSD comparing to the patients with normal ICP (right ONSD, OR: 5.2 mm, *P* = 0.001, Kappa: 0.67 and for left ONSD OR: 4.1 mm, *P* = 0.002, Kappa: 0.76). Additionally comparing OND of patients with high ICP with ones with normal ICP demonstrates the significantly higher OND in patients with high ICP (RT OND, OR: 1.15, *P* = 0.000 and for LT OND OR: 1.20, *P* = 0.000).

Considering 5.7 mm as the upper limit for normal ONSD, sensitivity, specificity, positive predictive value, negative predictive value, and accuracy validity of optic sonography in diagnosis of PPE are shown in Table 2.

Due to the lack of enough male subjects we could not assess the accuracy validity of ONSD measurement in detecting pseudopapilledema based on gender. Based on the measurements age did not affect accuracy validity of optic sonography for detecting pseudopapilledema (RT.ONSD sig: 0.497 and LT.ONSD sig: 0.21).

**Figure 1:**
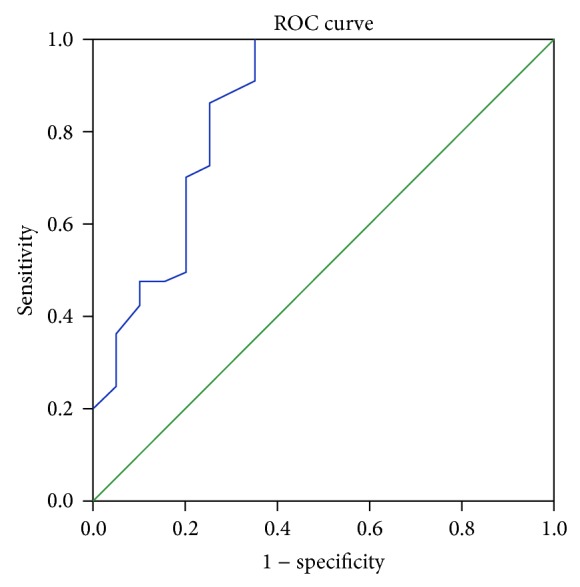
Roc diagram showing correlation of total ONSD and the CSF pressure.

The area under the curve (AUC) for RT.ONSD and LT.ONSD is 0.8 and 0.91 (*P* < 0.01 for AUC = 0.5), respectively. The best RT.ONSD cut-off value for detection of invasive ICP > 20 mmHg is 5.95 mm with 86% sensitivity and 70% specificity. The best LT.ONSD cut-off value for detection of invasive ICP > 20 mmHg is 5.86 mm. The sensitivity of this cut-off is 90% and the specificity is 80%. The best ONSD cut-off for both eyes together is 5.91 mm (sensitivity 86%, specificity 75%, AUC 0.85, *P* < 0.000) (Figure 1).

The best RT.OND cut-off for the detection of invasive ICP > 20 mmHg is 3.15 mm (sensitivity 78%, specificity 66%, AUC 0.8, *P* < 0.01). The best LT.OND cut-off for the detection of invasive ICP > 20 mmHg is 3.19 mm (sensitivity 85%, specificity 63%, AUC 0.74, *P* < 0.04). We also calculated OND cut-off for both eyes together that was 3.19 mm (sensitivity 82%, specificity 59%, AUC 0.77, *P* < 0.001).

## 4. Discussion

Our study demonstrates that optic nerve ultrasound with measurement of ONSD (optic nerve sheath diameter) is a highly accurate noninvasive technique for detection of intracranial hypertension. The literature, supporting our data, has reports of correlation between ONSD and intracranial hypertension among intracranial hemorrhagic patients [2] and based on considering 7.3 mm as the upper limit of normal ONSD [3]. Correspondingly some reports show that optic nerve ultrasound can be delightfully helping for ICP raising diagnosis when other imaging methods or invasive neurological testing is contraindicated in patients [4, 5].

Also in our research based on considering 5.7 mm as the upper limit of normal ONSD for detecting ICP more than 20 cm Hg, the sensitivity and negative predictive value of optic nerve ultrasound in diagnosis of pseudopapilledema for the right and left eyes were 100%. There are different reports for sensitivity and negative predictive values among literatures; Rajajee et al., by assessing ONSD in 56 patients with head trauma, intracranial hemorrhage, ischemic stroke, and cranial tumor, found that ICP raising correlates with ONSD in optic nerve ultrasound. With ROC curve and analysis they demonstrated that the optimal cut-off for ICP > 20 cm Hg is 4.8 cm for both eyes with 96% sensitivity and 94% specificity [6]. Moreover, Major et al. assessed ONSD in 26 patients. They showed that the optimal ONSD cut-off for increased ICP is 5 mm with 86% sensitivity and 100% specificity [7]. There are different sensitivity and specificity values for different cut-off chosen for ONSD demonstrating increased ICP among literatures [8, 9]. These different reports could be as a matter of fact that each research has different population with different epidemiologic features and also different number of subjects; furthermore choosing lower measures for ONSD cut-off in optic nerve ultrasound can affect the statistical values in this regard. To the authors' knowledge, there are not many reports for assessing a reliable ONSD cut-off among Iranian population [10]; also there are not many reports for assessing correlation between OND and ICP raising among literature which make our research novel. There are some reports among literature which chose their cut-off 5.7 mm in the same way as we did [11, 12]. On the other hand, there are many of reports that chose a lower cut-off which has a wide threshold among different researches from 4 mm to more than 5 mm [8, 9]. Besides we found some different data for ONSD and OND in detecting of raised ICP in comparison of left and right eye together that was not considered in other researches. All these different reports contribute to one similar conclusion; optic nerve ultrasound is a reliable diagnostic test for detecting raised ICP.

One limitation of our study was our sample size; there would be lower measure for our ONSD cut-off if we chose a larger sample size. Another limitation was that only suspected pseudotumor cerebri patients were included with normal neuroimaging and hence more female patients were included. We could not find any relationship between gender and ONSD because about 90% of our subjects were female. This issue could be considered for further researches that female's and male's ONSD cut-off could be different or not.

## 5. Conclusion

Our study demonstrated a close correlation between optic nerve sheath dilation on ocular ultrasound and evidence of elevated ICP with optic disk swelling. As mentioned before ONH swelling has many differential diagnoses but the critical one is true papilledema which could be a sign of raised ICP and a serious brain problem which need urgent intervention. Hence, clinicians must rule out pathological presentations that can be misinterpreted as swollen anomalous nerves which are called pseudopapilledema. Our study showed optic nerve sonography as a reliable diagnostic method in this regard for further usage.

## Figures and Tables

**Table 1:** Results (RT = right, LT = left, OND = optic nerve diameter, and ONSD = optic nerve sheath diameter).

	Number	Mean	Range
CSF pressure	32	25.87 ± 9.26	12–60
RT.ONSD	32	6.34 ± 1.02	3.22–8
RT.OND	28	3.49 ± 0.66	2.35–5
LT.ONSD	32	6.15 ± 0.84	4.35–8
LT.OND	28	3.44 ± 0.51	2–4.5

**Table 2:** Sensitivity, specificity, positive predictive value, negative predictive value, and accuracy validity of optic sonography in diagnosis of PPE.

	Sensitivity	Specificity	Positive predictive value	Negative predictive value	Accuracy validity
RT.ONSD	100	84	60	100	90
LT.ONSD	100	88	70	100	87
Both eyes together	100	83	55	100	86
